# Supplementary material for: Tocilizumab for the Treatment of Familial Mediterranean Fever—A Randomized, Double-Blind, Placebo-Controlled Phase II Study
Source: J Clin Med. 2022 Sep 13;11(18):5360. doi: 10.3390/jcm11185360 (PMC9500594; doi:10.3390/jcm11185360)
Supplement: Supplementary file 1 [file jcm-11-05360-s001.zip › jcm-1870947-supplementary.pdf]

Supplementary Appendix TOFFIFE trial

Content

1. Supplementary Table S1 Complete list of the 5 German centers with principle investigator 2

2. Supplementary Table S2 Tel Hashomer criteria (taken from: Livneh A, Langevitz P, Zemer D, et al. Criteria for the diagnosis of familial Mediterranean fever. Arthritis Rheum 1997; 40(10): 1879-85.) 2

3. Supplementary S3: Listing of in- and exclusion criteria: 2

4. Supplementary Table S4: Definition of the physicians' global assessment tool (PGA): 6

5. Supplementary Table S5: Diary: A German version of this diary was handed out to the patients, where they were asked to document their symptoms and NSAID intake every day during the last month 7

6. Supplementary Table S6: Mutations of the patients; patient number (1 = center Tuebingen, 2 = center Berlin, 3 = center cologne + randomization number) with randomized therapy and type of mutation 7

7. Supplementary figure S7: SAA (no exclusion of extreme values) 9

8. Supplementary Table S8: Absolute SAA levels over time (Week 0: Placebo n = 12, Tocilizumab n = 13 After Week 16: Placebo n = 3, Tocilizumab n=10, Change from Placebo to Tocilizumab (rescue arm) n = 4, Withdrawals until week 16: Pla n=4+1(One patient withdrew after having attended week 16), Tzc n=3) 10

9. Supplementary figure S9: Consort flow diagram 11

10. Supplementary Table S10: Listing of AEs from week 16: The rescue treatment group contains only patients who continued the study after week 16. 7 patients dropped out until week 16 (PBO=4, TCZ=3). One patient in the PBO group dropped out immediately after week 16. 4 Patients used the rescue option and changed from PBO to TCZ (see PBO/TCZ group). 12

11. Supplementary Table S11: Secondary endpoints showing no significant difference in the two arms (ANCOVA with baseline as covariate), abbreviations: VAS: visual analog scale, mm: millimeter, FFbH: Funktionsfragebogen Hannover (100% reflects normal function 13

12. Supplementary Table S12: Line listing of adverse events showing no difference and no new safety concerns 13

13. Supplementary Table S13: Comparison of Expected and Observed Study Results Concerning the Proportions of Successfully Treated Patients. Expected Results (week 16) Observed Results (week 16); Proportion of successfully treated patients: 14

1. **Supplementary Table S1 Complete list of the 5 German centers with principle investigator**

**Center**	**PI**
1. Tuebingen	Joerg Henes
2. Berlin	Martin Krusche/Eugen Feist
3. Cologne	David Kofler
4. Hamburg	Ina Koetter
5. Munich	Hendrik Schulze-Koops

2. **Supplementary Table S2 Tel Hashomer criteria (taken from: Livneh A, Langevitz P, Zemer D, et al. Criteria for the diagnosis of familial Mediterranean fever. Arthritis Rheum 1997; 40(10): 1879-85.)**

**Major Symptoms**	**Minor Symptoms**
Recurrent febrile episodes with serositis (peritonitis, synovitis or pleuritis)	Recurrent febrile episodes
Amyloidosis of AA type without a predisposing disease	Erysipelas-like erythema
Favorable response to regular colchicine treatment	FMF in a first-degree relative

Two or more major symptoms, or one major plus two minor symptoms, as listed below.

3. **Supplementary S3: Listing of in- and exclusion criteria:**

Inclusion Criteria

Subjects meeting all of the following criteria will be considered for admission to the trial:

- Age ≥ 18 years and written informed consent

- FMF according to the Tel Hashomer Criteria (see Appendix 14.4); with at least one heterozygous or homozygous mutation of the MEFV gene
- Inadequate response or intolerance to colchicine (inadequate response/intolerance: disease activity despite colchicine with at least 2 x 0.5 mg/day or intolerance to colchicine)
- Attack during the last 12 weeks, defined as episodes of fever and/or pericarditis and/or serositis and/or testis involvement and/or arthritis and/or erysipelas-like rash **and**
    - CRP > 0.5 mg/dl and/or ESR > 20mm/h and/or SAA > 10mg/dl
    - PGA >2
- Understand and voluntarily sign an informed consent document prior to any study related assessments/procedures.
- Ability to adhere to the study visit schedule and other protocol requirements.
- Females of childbearing potential (FCBP[1] A female of childbearing potential is a sexually mature woman who: 1) has not undergone a hysterectomy or bilateral oophorectomy; or 2) has not been naturally postmenopausal for at least 24 consecutive months (i.e., who has had menses at any time in the preceding 24 consecutive months) must agree:
    - to utilize two reliable forms of contraception simultaneously from heterosexual contact for at least 28 days before starting study drug, while participating in the study (including dose interruptions), and for 6 months after study treatment discontinuation and must agree to regular pregnancy testing during this timeframe
    - to abstain from breastfeeding during study participation and 6 months after study drug discontinuation.
- Males must agree
    - to use a latex condom during any sexual contact with FCBP while participating in the study and for 6 months following discontinuation from this study, even if he has undergone a successful vasectomy
    - to refrain from donating semen or sperm while on Tocilizumab/Placebo and 6 months after discontinuation from this study treatment.

- All subjects must agree to refrain from donating blood while on study drug and 6 months after discontinuation from this study treatment.
- All subjects must agree not to share medication.

Exclusion Criteria

Subjects presenting with any of the following criteria will not be included in the trial:

- Patient participating simultaneously in other clinical interventional trials
- Major surgery within 8 weeks prior to screening or planned major surgery within 12 months after randomization
- Transplanted organs (except corneal transplant performed more than 3 months prior to screening)

Exclusions Related to Prior or Concomitant Therapy

- Previous treatment with TCZ
- Treatment with glucocorticosteroide >10mg/day within 1 week; prednisolone ≤ 10mg/day can be given on a stable dose throughout the study
- Analgesic medication, other than paracetamol or ibuprofen or diclofenac or colchicine, which can be used at a stable dose throughout the study and/or for treatment of FMF attacks to the maximum allowed daily dose (paracetamole: 4000mg/day, ibuprofene: maximum 2400mg/day, diclofenac maximum 150mg/day; colchicine 12mg/day).
- Treatment with any investigational agent within 12 weeks (or 5 half-lives of the investigational drug, whichever is longer) of screening
- Treatment with Anakinra within the last 1 week prior to baseline (ptb), Canakinumab within the last 8 weeks prior to baseline
- Treatment with etanercept within 2 weeks; certolizumab pegol, abatacept or adalimumab within 6 weeks; golimumab and infliximab within 8 weeks ptb
- Rituximab within 24 weeks ptb
- Leflunomide within 12 weeks ptb (washout possible),
- azathioprine, cyclophosphamide within 12 weeks ptb

- Immunization with a live/attenuated vaccine within ≤ 4 weeks ptb
- Previous treatment with cell-depleting therapies, including investigational agents or approved therapies: anti-CD33, anti-CD52, anti-CD4, anti-CD5, anti- CD3 and anti-CD19
- Treatment with intravenous gamma globulin within 6 months of baseline
- Treatment with plasmapheresis within 6 months of baseline
- Any previous treatment with alkylating agents such as chlorambucil, or with total lymphoid irradiation

Exclusions Related to General Safety

- History of severe allergic or anaphylactic reactions to human, humanized, or murine antibodies
- Evidence of serious uncontrolled concomitant cardiovascular, nervous system, pulmonary (including obstructive pulmonary disease), renal, hepatic, psychiatric or gastrointestinal (GI) disease
- History of diverticulitis, diverticulosis requiring antibiotic treatment, or chronic ulcerative lower GI disease such as Crohn's disease, ulcerative colitis, or other symptomatic lower GI conditions that might predispose a patient to perforations
- Known active current or history of recurrent bacterial, viral, fungal, mycobacterial, or other infections (including but not limited to tuberculosis (TB) and atypical mycobacterial disease, hepatitis B and C, and herpes zoster, but excluding fungal infections of the nail beds)
- Any major episode of infection requiring hospitalization or treatment with IV antibiotics within 4 weeks of screening or oral antibiotics within 2 weeks of screening
- Active TB requiring treatment within the previous 3 years; patients should be screened for latent TB and, if positive, treated according to local practice guidelines prior to initiating TCZ treatment; patients treated for TB with no recurrence within 3 years and patients treated for latent TB within 3 years are eligible.
- Primary or secondary immunodeficiency (history of or currently active)
- Evidence of malignant disease or malignancies diagnosed within the previous 5 years (except basal and squamous cell carcinoma of the skin or carcinoma in situ of the cervix uteri that have been excised and cured)

- FCBP who are not willing to use an effective method of contraception, such as condom, sterilization during the study and for a minimum of 6 months after study drug therapy and breast-feeding females
- Pregnant women
- Males of reproductive potential who are not willing to use an effective method of contraception, such as condom, sterilization, throughout study and for a minimum of 6 months after study drug therapy
- History of alcohol, drug, or chemical abuse within 1 year prior to screening

Laboratory Exclusions (at Screening)

- Serum creatinine >1.4 mg/dL in female patients and >1.6 mg/dL (in male patients
- ALT or AST > 2 ×ULN
- Total bilirubin > 2 x ULN
- Platelet count < 100 × 109/L
- Hemoglobin < 8.5 g/dL
- White blood cells < 3.0 ×109/L
- Absolute neutrophil count < 2.0 × 109/L
- Absolute lymphocyte count < 0.5 × 109/L
- Positive hepatitis B surface antigen, anti-HBc, HIV or hepatitis C antibody

4. Supplementary Table S4: Definition of the physicians' global assessment tool (PGA):

		0 = No	1 = minimal<br>(max 7 days)	2 = mild<br>(max 14 days)	3 = moderate<br>(max. 21 days)	4 = severe (whole month)

1	Thoracic pain<br>(during the last 4 weeks)					
2	Abdominal pain					
3	Arthralgia					
4	Arthritis					
5	Erythema					
6	Fever >38°C					
	**Sum:**					

5. **Supplementary Table S5: Diary: A German version of this diary was handed out to the patients, where they were asked to document their symptoms and NSAID intake every day during the last month**

Day	Date	Fever > 38°	Abdominal Pain	Thoracic pain	Arthralgia	Erythema	Paracetamol 1 dash = 500mg	Ibuprofen, 1 dash = 600mg	Diclofenac 1 dash = 50mg
									…

6. **Supplementary Table S6: Mutations of the patients; patient number (1 = center Tuebingen, 2 = center Berlin, 3 = center cologne + randomization number) with randomized therapy and type of mutation**

**Pat.**	**therapy**	**MEFV mutation:**	**1 = heterozygous<br>2 = homozygous**
1-01	Placebo	M694V + R761H	1
1-02	Placebo	M694V + V726A	1

Pat.	therapy	MEFV mutation:	1 = heterozygous<br>2 = homozygous
1-03	Tocilizumab	M694V	2
1-04	Tocilizumab	M680I + M694V	1
1-07	Tocilizumab	M694V	2
1-08	Placebo	M680I	1
1-09	Tocilizumab	M694V	1
1-10	Placebo	M694V	2
1-11	Tocilizumab	M680L	1
1-12	Placebo	M694V + V726A	1
1-14	Placebo	M694V	2
1-16	Placebo	P369S + R408Q	2
1-20	Tocilizumab	M694V	1
1-21	Placebo	A103T + L695A	1
2-19	Placebo	M694V	2
2-23	Tocilizumab	M694V	2
2-24	Placebo	M694V	2
2-25	Tocilizumab	V726A	2
2-26	Placebo	M649V	2
2-28	Tocilizumab	M694V	2
2-30	Tocilizumab	V726A + M694I	1
3-13	Tocilizumab	M694V	2
3-18	Tocilizumab	M680I + M694V	1
3-22	Tocilizumab	M694V	2
3-31	Placebo	M6802A	2

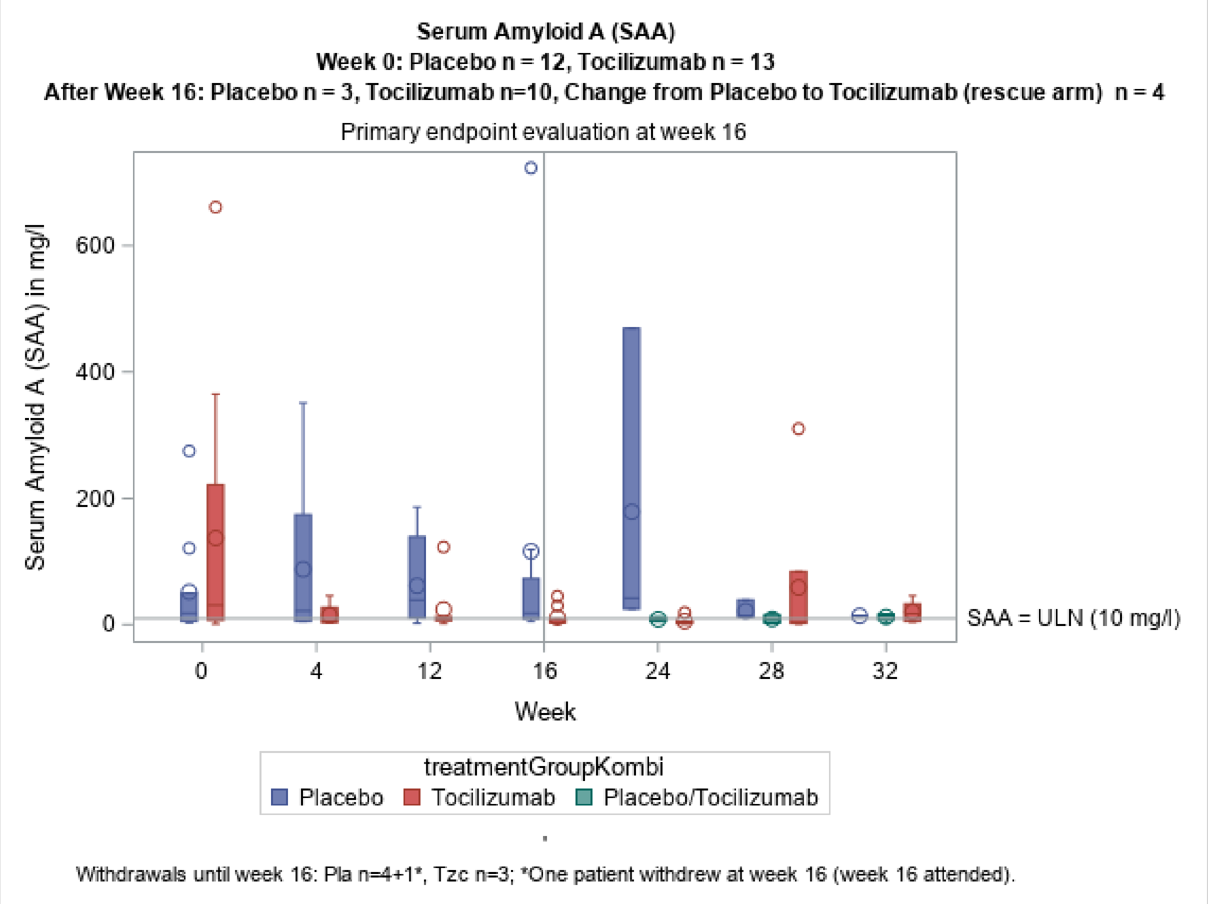

7. Supplementary figure S7: SAA (no exclusion of extreme values)

8. **Supplementary Table S8: Absolute SAA levels over time (Week 0: Placebo n = 12, Tocilizumab n = 13 After Week 16: Placebo n = 3, Tocilizumab n=10, Change from Placebo to Tocilizumab (rescue arm) n = 4, Withdrawals until week 16: Pla n=4+1(One patient withdrew after having attended week 16), Tzc n=3)**

Treatment group	Week	N	Missings	Maximum	Q3	Median	Q1	Minimum
Placebo	0	11	1	275	50	18	5	3
	4	7	5	351	174	22	5	5
	12	7	5	186	139	39	11	2
	16	8	4	723	73	18	9.5	5
	24*	3*	0*	469	469	42	25	25
	28	3	0	39	39	14	12	12
	32	1	2	14	14	14	14	14
Placebo/ Tocilizumab	24	2	2	12	12	8.5	5	5
	28	4	0	18	15.5	8	2.5	2
	32	4	0	17	17	14.5	7.5	3
Tocilizumab	0	12	1	661	221.5	31	7	1
	4	4	9	46	27	5.5	3	3
	12	7	6	123	13	8	6	1.8
	16	9	4	45	9	3	3	2
	24*	6*	4*	19	5	3.5	2	1
	28	7	3	310	84	4	2	1
	32	8	2	46	33	17.5	5	4

*Patients were offered the rescue medication TCZ after week 16, 4 patients changed from the PBO group to TCZ.

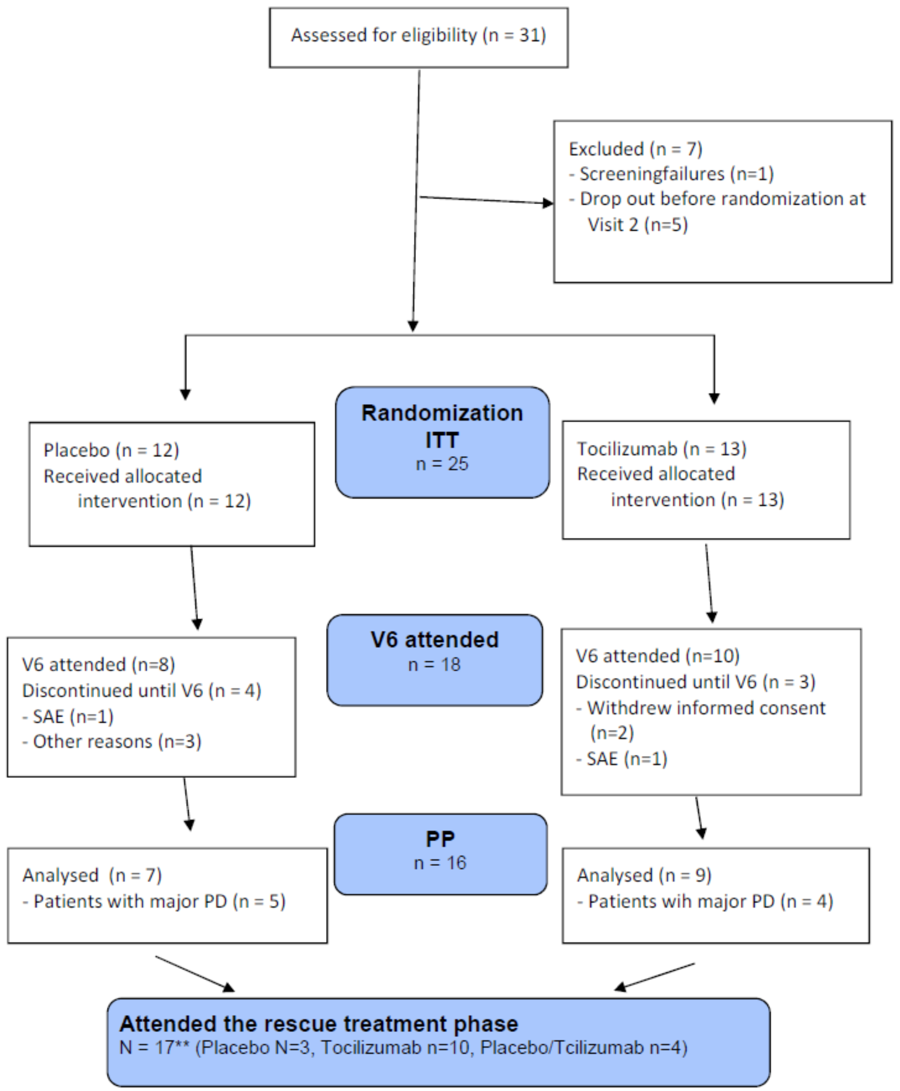

9. Supplementary figure S9: Consort flow diagram

*** One patient dropped out after having completed the visit 6 assessments.*

10. **Supplementary Table S10: Listing of AEs from week 16: The rescue treatment group contains only patients who continued the study after week 16. 7 patients dropped out until week 16 (PBO=4, TCZ=3). One patient in the PBO group dropped out immediately after week 16. 4 Patients used the rescue option and changed from PBO to TCZ (see PBO/TCZ group).**

	**Rescue treatment group**			
**AEs from week 16 until end of study**	**PBO (N = 3) (%)**	**TCZ (N = 10) (%)**	**PBO / TCZ (N = 4) (%)**	**Total (N = 17) (%)**
Patients with AEs	2 (66.7%)	5 (50.0%)	4 (100.0%)	11 (64.7%)
Number AEs	5	18	12	35
No category	0	1 (5.6%)	0	1 (2.9%)
Infection	1 (20.0%)	2 (11.1%)	3 (25.0%)	6 (17.1%)
Joint complaint	1 (20.0%)	1 (5.6%)	1 (8.3%)	3 (8.6%)
FMF flare	0	10 (55.6%)	0	10 (28.6%)
Skin disorder	1 (20.0%)	3 (16.7%)	2 (16.7%)	6 (17.1%)
Cardiac	0	0	0	0
Gastroenterology	0	1 (5.6%)	1 (8.3%)	2 (5.7%)
Coagulopathy Bleeding	0	0	5 (41.7%)	5 (14.3%)
Other	2 (40.0%)	0	0	2 (5.7%)

11. **Supplementary Table S11: Secondary endpoints showing no significant difference in the two arms (ANCOVA with baseline as covariate), abbreviations: VAS: visual analog scale, mm: millimeter, FFbH: Funktionsfragebogen Hannover (100% reflects normal function)**

**Secondary endpoints**	**Placebo (N = 12)** Median (range)	**Tocilizumab (N = 13)** Median (range)	**Total (N = 25)** Median (range)	**P value**
VAS physician, baseline, in mm	66.5 (0.0; 90.0)	60.0 (24.0; 82.0)	65.0 (0.0; 90.0)	
VAS physician, week 16, in mm	38.0 (0.0; 77.0)	29.5 (0.0; 62.0)	34.0 (0.0; 77.0)	**0.18**
VAS patient, baseline, in mm	47.0 (0.0; 77.0)	58.0 (15.0; 88.0)	55.5 (0.0; 88.0)	
VAS patient, week 16, in mm	38.5 (0.0; 78.0)	33.0 (0.0; 78.0)	33.5 (0.0; 78.0)	**0.91**
FFbH, baseline, in %	88.9 (8.3;100.0)	91.7 (66.7; 100)	91.7 (8.3; 100)	
FFbH, week 16, in %	86.6 (58.8; 100)	91.7 (80.6; 100)	88.9 (58.8; 100)	**0.11**

12. **Supplementary Table S12: Line listing of adverse events showing no difference and no new safety concerns**

	**Randomized treatment group**		
**AEs until date of V6 / week 16**	**PBO (N = 12)**	**TCZ (N = 13)**	**Total (N = 25=**
Patients with AEs	8 (66.7%)	12 (92.3%)	20 (80%)
Number AEs	22	51	73
No category	0	0	0
Infection	5	10	15

AEs until date of V6 / week 16	Randomized treatment group<br>PBO<br>(N = 12)	 TCZ<br>(N = 13)	 Total<br>(N = 25=
Joint complaint	1	6	7
FMF flare	4	10	14
Skin disorder	2	6	8
Cardiac	3	2	5
Gastroenterology	2	9	11
Other	5	8	13

13. Supplementary Table S13: Comparison of Expected and Observed Study Results Concerning the Proportions of Successfully Treated Patients. Expected Results (week 16) Observed Results (week 16); Proportion of successfully treated patients:

Expected Results (week 16)	Observed Results (week 16)
Proportion of successfully treated patients:	Proportion of successfully treated patients (missing value imputation: non-responders):
TCZ-group: 83% (planned n=15)	TCZ: 15.4% (95%-CI: 2% - 45%), n=13 (imputation n=4)
PCB-group: 40% (planned n=15)	PCB: 0% (95%-CI: 0% - 22%), n=12 (imputation n=4)
alpha=0.2 (two-sided)	p=0.089

The result of the final analysis is statistically significant. The results of the sensitivity analyses and the PP-analyses and the analysis of the secondary endpoints also suggest a superiority in the efficacy of the TCZ-treatment compared to Placebo-treatment.
